# Koller of Austria

**Published:** 1889-06

**Authors:** 


Koller of Austria, the discoverer of Cocaine, has removed from Vienna to New York City.

